# Changing the Sickle Cell Nutrition Integration Narrative: Qualitative Perspectives From Sickle Cell Service Users/Carers About Nutritional Care

**DOI:** 10.1111/jhn.70303

**Published:** 2026-07-14

**Authors:** Claudine Matthews, Adrian Brown, Michelle Hawkins, David Smith

**Affiliations:** ^1^ Anglia Ruskin University, Faculty Health, Medicine and Social Care, Bishops Hall Chelmsford UK; ^2^ Division of Medicine University College London London UK; ^3^ Centre for Obesity Research University College London (UCL) London UK; ^4^ Bariatric Centre for Weight Management and Metabolic Surgery University College London Hospital NHS Trust (UCLH) London UK

**Keywords:** nutrition integration, nutritional care, perspectives, qualitative, Sickle cell disease, sickle cell service users/carers

## Abstract

**Introduction:**

Nutrition is not currently integrated into standard care provision in sickle cell disease (SCD) impacting patients experience, access and outcomes of nutrition. The lack of understanding of the nutritional needs in SCD and its impact on patient outcomes requires exploration to integrate nutrition into clinical practice. However, there is a paucity of qualitative studies conducted with sickle cell service users/carers to explore their views, knowledge and experiences of nutritional care and the influencing factors affecting nutrition integration in SCD care.

**Methods:**

An independent focus group involving SCD service users and carers formed part of a larger four phased participatory sequential Learning Alliance study conducted between March and December of 2020. Purposive sampling was used to recruit suitable adult sickle cell service users/carers (*n* = 11) to participate in this UK based study.

**Results:**

Four main themes were generated including 1) The invisibility of SCD, 2) Under ‐recognised importance of nutrition, 3) Lack of priority to nutrition and 4) Multi‐level factors affecting nutrition and service provision that underscore key knowledge and care gaps in addition to an array of influencing factors affecting nutrition integration in SCD. Moreover, these themes reflect the complexity of sickle cell nutrition and the impact of the lack of nutrition integration on SCD patients access and outcomes of nutrition.

**Conclusion:**

The main findings provide evidence of the nutrition knowledge and care gaps in SCD and the need to address the under‐recognition and lack of priority to nutrition, driving the lack of nutrition integration and poor patient outcomes in SCD.

## Summary

- A qualitative participatory learning alliance study, using a socio‐ecological lens was conducted to identify the multiple influencing factors affecting nutrition integration in SCD by exploring the views, knowledge and lived experience of 11 sickle cell service users/carers about nutritional care in the UK.
- Currently nutrition is not part of standard care provision in SCD evident in the lack of research on the nutritional management of people with SCD, confounding the lack of nutrition service provision and policy and practice to support the nutritional management in this patient population.
- The lack of nutrition service provision reflects key knowledge and nutritional care gaps impacting the experience, access and outcomes of sickle cell patients to tailored nutritional care in the NHS. This lack exists, despite the existing scientific literature identifying a direct link between the main clinical features of SCD and the chronic nutritional problems linked to SCD, that requires nutritional management.

## Graphical abstract

This novel qualitative study provides primary data that reflects the sickle cell service user/carer voice about the changes needed to redefine and integrate nutritional management in standard SCD care.

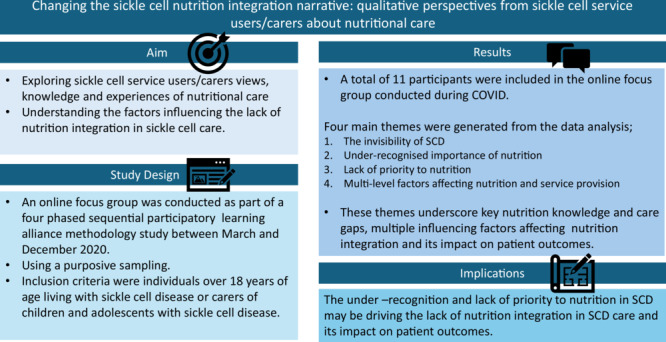

## 1 Introduction

Sickle cell disease (SCD) is a genetically inherited red blood cell disorder, recognised as a global public health condition [1] with an estimated global prevalence of 7.7million in 2021 [2]. In the UK, SCD has an estimated prevalence of 17,500 people and is deemed to be the fastest growing and most common genetic disorder [3]. However, compared to other genetic disorders with a lower overall prevalence such as cystic fibrosis where nutrition is firmly integrated into standard care [4], integrated sickle cell nutrition is lacking. Despite multiple studies identifying the need for nutrition to be part of standard clinical care in SCD [5, 6, 7, 8, 9] in addition to clear causal links between the clinical features of SCD and nutrition [10], it remains an often overlooked and forgotten aspect of care provision [11]. This persistent lack of nutrition service provision available to sickle cell patients has the potential to drive health inequalities, affecting sickle cell patients' experience, access and outcomes of nutrition [12].

Presently, in addition to the lack of nutrition policy and practice in sickle cell nutrition, there is a lack of research on how to integrate nutrition into standard care in SCD and an array of influencing factors affecting nutrition and service provision within the UK, since very little is known about the current nutrition landscape in SCD care provision. Several psychosocial factors [13] and social determinants of health [14, 15, 16] have been identified to impact on the health and wellbeing outcomes of people living with SCD, however these factors have not formerly been examined in the context of its impact on the nutritional needs, risks and challenges of people living with SCD.

Anecdotally, nutrition is reported as the most common question asked by sickle cell patients, signalling the need for more to be done to address the invisibility of nutrition and the growing health inequality linked to the lack of nutrition service provision available to sickle cell patients. There is a lack of qualitative studies conducted with sickle cell service users/carers and providers in sickle cell nutrition. Thus, more effort is needed to acknowledge the patient voice in calling for the integration of nutrition into standard care in SCD. Therefore, the aim for this study was to identify the views, knowledge, and experiences of sickle cell service users/carers (SCSU‐C) and the socio‐ecological influencing factors affecting nutrition integration in SCD, with the overall aim of looking at how to integrate sickle cell nutrition within clinical practice.

## 2 Material and Methods

### 2.1 Design

This study design includes an independent focus group as part of a larger sequential participatory mixed methods learning alliance methodology [17, 18, 19] involving adult sickle cell service users and carers.

### 2.2 Sampling and Recruitment

The recruitment process involved purposive sampling with adults (aged over of 18 years) living with sickle cell or adults' carers (aged over 18 years) looking after children or adolescents living with SCD.

Recruitment involved the help of gatekeepers and placement of adverts in the local hospital clinical areas with details of the study and the contact details of the research team alongside promotion by the Sickle Cell Society (SCS), the UK national sickle cell charity, on their website. Interested participants then emailed or called the research team to express their interest in participating in the study. Based on whether they met the eligibility criteria, all the eligible participants (*n* = 11) were then emailed a copy of the participant information sheet (PIS) and consent form by the research team outlining the aim, requirements and relevant dates for the study and were given the opportunity to ask any questions pertaining to the study and their involvement with the researcher. A total of 11 participants contacted the research team and deemed eligible and were asked to return a signed digital copy of the consent form on the day of the focus group indicating their ongoing willingness to participate in the study.

The focus group was conducted online using Zoom (Zoom Communications Inc) as the preferred digital platform. There was a total of 11 participants in the focus group that lasted 120 min allowing time for a comfort break.

### 2.3 Data Collection

#### 2.3.1 Design of Questionnaire for Focus Group

The focus group topic guide design was informed by the available literature on sickle cell nutrition [10] and developed to explore four key areas of interest which were 1) the medical management of SCD 2) the nutritional management of SCD 3) the socio‐ecological factors affecting nutrition and 4) the facilitators to address these socio‐ecological factors.

#### 2.3.2 Semi‐Structured Questionnaire

The topic guide consisted of 10 semi‐structured questions (supporting information S1) taking a comprehensive approach to the data collection in order to identify and understand participants views, knowledge and experiences of the medical and nutritional management in SCD. In addition, a socio‐ ecological lens was applied to identify and understand the influencing factors affecting the lack of nutrition integration in SCD and facilitators to address these influencing factors. The topic guide was designed by the researcher CM with support from DS and MH.

### 2.4 Data Analysis

The data was collected and recorded using zoom as the chosen digital platform, transcribed verbatim, then analysed using the six phase Braun and Clarke [20, 21] thematic process that allowed for full immersion in the data set accommodating for the iterative nuances and interpretations of the data during and after the data analysis process. CM was the main coder maintaining open dialogue with DS and MH throughout the six phases to mitigate for discrepancies in the analysis process. Phase one and two involved the familiarisation of the whole data set to identify initial insights and ideas before identifying initial codes by CM which was transferred to an excel spread sheet and shared with DS and MH for review and feedback. Phase three and four involved generating initial themes by CM using the specific code categories identified and agreed by DS and MH before using these codes to develop the study themes checked against the central organising concepts of each theme. Phase five and six facilitated the defining and refining of the themes by CM, as reviewed by DS and MH, which remained unchanged as it supported the project aims and accounted for any unexpected findings to emerge from the codes and themes developed.

## 3 Results

### 3.1 Participants

Eleven SCSU‐Cs (*n* = 9 Females, one is a carer, *n* = 2 Males) participated in this independent focus group which was held online in March 2020. Despite this being the start of the COVID lockdown, participant perspectives or increased caregiver obligations did not appear to impact participant engagement. Although the specific demographic data for the SCSU‐Cs such as employment status, qualifications and age, were not collected to promote participant engagement in the study, the data collection allowed for the participants to reflect on how these factors impacted on their knowledge, views and experiences of the medical and nutritional management of SCD.

### 3.2 Key Themes

The analysis of focus group findings from the SCSU‐C participants contributed to the development of the four main themes which were: 1) The invisibility of SCD, 2) Under‐recognised importance of nutrition, 3) Lack of priority to nutrition and 4) Multi‐level factors affecting nutrition and service provision. These are described in more detail in Table 1. In addition, there were key subthemes as reported below, with some occurring across all four themes relating to poor knowledge and awareness of nutrition.

**Table 1:** Definition of the four key themes.

Theme	Definition
**1. Invisibility of SCD**	Theme 1 evidence the disparities and confounding factors influencing the experiences and medical management of SCSU‐Cs of care provision in SCD, reflecting the health inequalities associated with SCD as a marginalised patient population.
**2. The under‐recognition of the importance of nutrition**	Theme 2 reflects the under‐recognition of the array of nutritional needs, challenges and risks experienced by people living with SCD and reflects the impact of poor knowledge and understanding of the link between the medical management and the nutritional management of SCD, and how these impact on the patient's experience, access and outcomes of nutrition in SCD.
**3. The lack of priority to nutrition**	Theme 3 reflects the under prioritisation of nutrition in SCD, evident in the paucity of nutrition resources and poor knowledge of nutrition and reflects the limited understanding of optimum nutrition, the benefits of nutrition in SCD healthcare provision and the need for education and training in nutrition in SCD to be tailored to the unique needs of people living with SCD.
**4. Multi‐level factors affecting nutrition and service provision**	Theme 4 thus provides an overview of the range of knowledge and care gaps necessitating a population level, whole systems approach to improve nutrition service provision in SCD, to address the effects of the marginalisation of SCD and the growing health inequalities associated with the medical and nutritional management of people living with SCD.

Theme 1 **– Invisibility of SCD –** *poor knowledge and awareness, management and treatment*.

*Theme 1 related to the impact of the invisibility of SCD and nutrition on the level and quality of care, management and treatment available to people living with SCD when accessing care in the NHS. This was confounded by poor knowledge and awareness of nutrition driving service users and carers to have to self‐research their knowledge of nutrition*.

### 3.3 Subtheme 1 – Poor Knowledge and Awareness

From the focus group discussions, SCSU‐Cs expressed that they had a lack of knowledge and awareness of sickle cell and nutrition which was reflected in the level of nutrition care provision available to them. They reported to have to self‐research what they wanted to know about nutrition despite nutrition being central to them ‘*staying well’* as reflected in this comment – *'nutrition for me I think plays a major part in staying well… I've kind of looked up myself – I'll go and do this research'* (SU‐C 6). This need to self‐research information about sickle cell nutrition was echoed in this comment – *'it really is about doing your own research and understanding'* (SU‐C, 7).

In addition, this participant highlighted that misinformation about nutrition was a concern, with one SCSU‐C explaining their interaction with a health visitor about not giving their child meat in their normal diet – *'We were not eating meat at the time, and we got a lot of flak for that. The health visitor used to say, you're going to make your child malnourished if you don't give them meat…it didn't change my mind because I had to do my own research.'* (SU‐C 8)

### 3.4 Subtheme 2 – Management and Treatment

The medical management of SCD needs to consider the additional medical needs sickle cell patients may experience as a consequence of growing older, expressed by one of the SCSU‐C participants – *'I have different medical needs as I get older, I find that I get more medical needs because as we're living longer with SCD, we also have age related issues to deal with…I have been a guinea pig all my life because of my age*.' (SU‐C 1)

The need for individualised care was highlighted by participants as important with this SCSU‐C highlighting the differences that exist between their own experiences of their sickle cell related problems depending on their genotype and phenotype and the need for each patient to be treated as an individual – *'A lot of us have differences between us – sickle cell patients have different phenotypes and genotypes – responsible for the variation and this can be present in families as well*.' (SU‐C 7)

Theme 2 – **Under‐recognised importance of nutrition –** *lack of inclusion/discussion of nutrition in standard care, poor quality of nutritional management, nutritional risk, nutritional challenges*.

*Theme 2 relates to the under‐recognition of the importance of the nutritional needs, risks and challenges of sickle cell patients, which are increased by the lack of understanding of the causal link between the clinical features of SCD and the nutritional implications of SCD. This inadvertently drives the lack of inclusion/discussion of nutrition in standard care, the lack of nutritional management and under recognition of the nutritional risks and challenges in SCD*.

### 3.5 Subtheme 1 – Lack of Inclusion/Discussion of Nutrition in Standard Care

The lack of inclusion/discussion of nutrition in standard care in SCD amongst healthcare professionals was apparent in the following comments expressed by a number of SCSU‐Cs about the lack of opportunity to speak about nutrition *'we never have a conversation like you have heard from so many others, we never have a conversation about nutrition*.' (SU‐C 9)

I feel like there needs to be a bit more discussions on nutrition… and that discussion is never had with my consultants or nutritionist that come around…a lot of discussions about you have to eat more, but never more of what, so yeah.

(SU‐C3)

– I think it's something that can be brought up a lot more cos for me for a lot of years it wasn't really addressed – definitely something that should be more spoken about.

(SU‐C 10)

it does need to be a big conversation.

(SU‐C 4)

Similarly, this participant highlighted that they have never directly been asked about their nutritional needs in their medical consultations *…I've never directly been asked about my nutrition and how I eat…* (SU‐C 6).

### 3.6 Subtheme 2 – Poor Quality of Nutritional Management

The need for better quality of nutritional management in SCD was apparent in the comment expressed by a SCSU‐C as they called for wider involvement of sickle cell healthcare professionals, a more comprehensive focus to nutrition and earlier nutritional care as explained in this comment *'in general there needs to be a bigger conversation from initial doctors to our consultants…the biggest like nutritional conversation I've ever had with any of my doctors was you need to drink more water…'there needs to be a bigger conversation because that would have been a lot more beneficial’* (SU‐C 10).

This participant expressed the need for clearer nutritional conversations to happen earlier rather than later *'The conversation needs to happen earlier, and continue to happen, so the reinforcement of the message of good health and nutrition, just as they reinforce you must take your folic acid and you must take your penicillin, it should be reinforced that you should be eating*.' (SU‐C 6).

### 3.7 Subtheme 3 – Nutritional Risks

The lack of nutritional management available to sickle cell patients added to the nutritional risks of this patient population evidenced in the unexpected diagnosis of a SCSU‐C with osteoporosis, as reported in the comment *'I for instance was diagnosed with osteoporosis recently and that was a bit of a shock to me and then they've now said oh yeah, we are going to start treating it now – oh well why wasn't it tracked before we got to this level…*.' (SU‐C 2).

The late diagnosis of nutritional problems in people living with SCD, was an important finding of the study, uncovering the potential risks for many sickle cell patients across the life course evident in the following comment from this SCSU‐C who is a sickle cell patient carer – *'…she asked me what his Vitamin D is like and I said well, I don't know. So, she said – ‘you're saying that no one has ever checked it’ and I said yeah, no one has ever checked his vitamin D – so we went to see her she asked me the question we were able to get a blood test for him and absolutely his vitamin D was like nothing*.' (SU‐C 11). The comment expressed by a SCSU‐C reflected the lack of value ascribed to the role of nutrition in SCD and the preference for the medical management over the nutritional management in SCD *– 'because their role is not to deal with nutrition but it's to deal with medication, pharmaceutical and stuff*.' (SU‐C 1).

### 3.8 Subtheme 4 – Nutritional Challenges

A number of health‐related factors that may contribute to the nutritional challenges faced by sickle cell patients were identified in the comments by these SCSU‐Cs,

we have a lot of inflammation in our bodies and various other antioxidants enhancing blood flow – medicating yourself on a day‐to‐day basis with foods that you can eat and try to maintain a decent level of wellbeing so when the inevitable crisis come, it is not so severe as it may be.

(SU‐C 7)

so my consultant told me that I was meant to be on a high protein high carb diet – so I tried it for a while, and it worked – but then I questioned why he didn't tell me earlier…because he only told me 2–3 years ago – it was just like kind of a bypass comments.

(SU‐C 4)

A further factor identified as a nutritional challenge experienced by sickle cell patients was appetite suppression, evident in the comments by these SCSU‐Cs,

when I'm ill food isn't really an interest to me.

(SU‐C 2)

I don't tend to eat when I am unwell, I don't eat when I am in hospital, if I do eat it's because I am getting better.

(SU‐C 6)

I know that for me I don't eat well at all when I am in hospital – the free meals in hospital doesn't really do anything for me….I myself find that nutrition is very important for my health, even in terms of mental health nutrition has been a massive part of my mental health.

(SU‐C 3)

A number of system related factors that may contribute to the nutritional challenges faced by sickle cell patients were identified in the following SCSU‐C comments,

it's just not factored, not factored at all into the I guess um medical evaluation of someone with sickle cell' …so the system is just not considerate whatsoever…, 'they push nutrition to the side, and it's not considered.

(SU‐C 7)

I think when you are looking at sickle patients in a more holistic way if the clinical standards are now saying that every patient should be entitled to a psychologist and pain therapist, or physiotherapist then why can't we also have nutrition as part of that and the evidence should also support that….

(SU‐C 9)

Theme 3 **‐ Lack of priority to nutrition –** *Poor knowledge and awareness, optimum nutrition, benefits of nutrition, inequalities in nutrition, education and training*.

*Theme 3 related to the lack of priority given to nutrition which was evident in the lack of nutritional care, poor nutrition knowledge, awareness and resources, the limited understanding of optimum nutrition in SCD and the lack of tailored nutrition education and training for both sickle cell patients and providers*.

### 3.9 Subtheme 1 – Poor Nutrition Knowledge, Awareness and Education and Training

The comments from these SCSU‐C participants highlighted the need for more education and training to be made available to sickle cell healthcare professionals including doctors and nurses that includes sickle cell nutrition as ‘nutrition is paramount’ in the management of SCD,

I believe sickle cell is not recognised as it should be…we are campaigning for doctors and nurses to know about basic nutrition apart from the other things. We believe that it's something that they should know you know when treating us and not just dealing with our medication that has side effects causing other issues, meaning more medication. Nutrition is paramount.

(SU‐C 1)

so in terms of doctors, I don't know if they have that much training on nutrition for people with SCD because you see you are deficient in a lot of things.

(SU‐C 11)

Well for me, most of the time I find that I know more than the doctors.

(SU‐C 2)

### 3.10 Subtheme 2 – Optimum Nutrition

**Figure 1:**
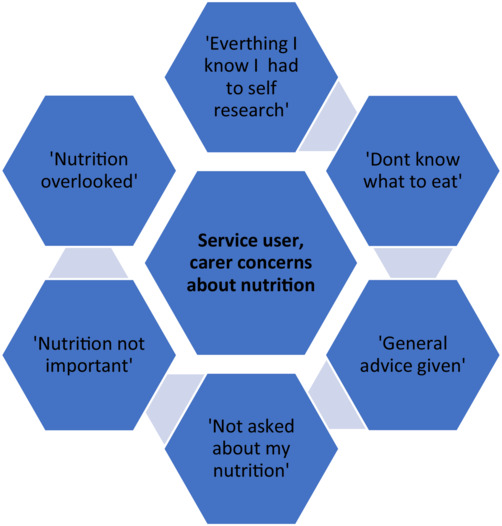
SCSU‐C concerns about the lack of nutrition service provision.

Figure 1 below illustrates the concerns SCSU‐Cs had about nutrition and not knowing what to eat, however it was apparent from the comment by one SCSU‐C that optimum nutrition may be understood in a limited way, viewed as an ideal nutritional goal which may not be achievable when they have low energy levels and not able to prepare food and vulnerable to making less than ideal food choices: *'so for me optimum nutrition is an ideal that is not necessarily something I can keep up with all the time because when you are not feeling well or have no energy to prepare food and anything else you just grab the nearest thing you can…*.' (SU‐C 2)

### 3.11 Subtheme 3 – Benefits of Nutrition

The benefit of nutrition in the management of SCD was explained in the comment by a SCSU‐C for whom nutrition was their main management option for their SCD and an essential lifeline to keep well, alongside the support of family members *– 'in the management of my sickle it's been central – I'm not on any medication so that's my medical position I do not take a fixed medicine except penicillin when unwell so outside of that this is how I was raised; my mother was a paediatric nurse*.' (SU‐C 9).

A SCSU‐C expressed the need for more proactive care with regular access to dietitians or nutritionists as a key part of prevention – *'having a more proactive approach…maybe if we had regular meetings with a nutritionist or a dietitian it wouldn't take me having the condition in the first place – I would have already you know prevented it before it happened'* (SU‐C 5). Furthermore, this SCSU‐C explained how they were fed via a tube early in their life because their nutrition was not very good and kept going into crisis – *'early in my life I had PEG feeding because my nutrition wasn't very good, so I kept going into crisis'* (SU‐C 4).

Theme 4 **– Multi‐level factors affecting nutrition and service provision –** *bio‐psychosocial factors, need for earlier education/knowledge, complexity of nutrition, infrastructure and systems, policy and practice*.

*Theme 4 related to the complexity of sickle cell nutrition and the multiple influencing factors affecting sickle cell patients' nutritional needs, risks and challenges. It also included the myriad of factors that needed to be considered when planning nutrition service provision in SCD, tailored to the specific nutritional needs of the sickle cell patient population*.

### 3.12 Subtheme 1 – Personal: Bio‐Psychosocial Factors

Several bio‐psychosocial factors were identified as personal influences affecting the nutritional needs, risks and challenges of sickle cell patients highlighted in these comments from two SCSU‐Cs – *'I am also guilty of comfort eating and that also applies when I am not feeling well'* (SU‐C 5) and *'Sometimes when I am really moody then I would tend to eat stuff that I naturally wouldn't eat'* (SU‐C 9).

Family related factors were highlighted in the following comment from a SCSU‐C demonstrating how the lethargy and tiredness commonly experienced by sickle cell patients affected their nutritional needs, risks and challenges and how reliant they were on family members for support on these occasions – *'I suffer with lethargy and tiredness, and I don't feel like cooking everyday…I'm really reliant on my family at home to do a lot of the cooking you know… my daughter keeps things chopped up in the fridge so its easily accessible'* (SU‐C 1).

In addition, the working pattern and environment of sickle cell patients may also influence what and when they are able to eat and the availability of healthy options as expressed in the comment by this SCSU‐C – *'Because of my job with long hours, I have most of my meals through work, for my breakfast, lunch and dinner, as a result I eat what's available there'* (SU‐C 5).

### 3.13 Subtheme 2 – Interpersonal Factors: Need for Earlier Education/Knowledge

The following comment from a SCSU‐C expressed some of the challenges associated with the timing of providing nutrition knowledge and education to sickle cell patients and how this is able to reinforce key health practices and become second nature *– 'So if the conversation happens prior to being hospitalised, then you kind of, you know like the more you hear something, the more it sinks in and becomes second nature. Then you are already aware of the things that you should be eating'* (SU‐C 6).

### 3.14 Subtheme 3 – Institutional Factors: Complexity of Nutrition

One of the SCSU‐C participants suggested having joint clinics for both paediatric and adult sickle cell patients providing a joint model of care allowing for the nutrition education of nurses and doctors whilst also enhancing their skill – *'well I think joint clinics would be great for both paediatric and adult patients…just sort of have that joint model of care, but we also educating either the nurses or the doctors and enhancing their skills as well'* (SU‐C 9). Furthermore, in the context of nutrition service provision, a SCSU‐C highlighted the need for education to be provided to healthcare leaders from the top level – '*I think you need to educate people from the top level'* (SU‐C 2).

### 3.15 Subtheme 4 – Community and Policy Factors – Infrastructure and Systems

The lack of nutrition resources and information available to sickle cell service users and providers in the context of the community was highlighted by this participant – *'I think it boils down to availability of the information'* (SU‐C 6). Similarly, one SCSU‐C explained the challenges with cost, affordability and availability of heathy food options in the local areas where people living with SCD live – *'there is not much in the way of healthy food options like in my surrounding areas there's a lot of chicken shops* – *like whole foods is a very expensive shop'* (SU‐C 6). In addition, a SCSU‐C expressed the value and importance of sickle cell patients having access to cooking workshops, similar to what is being done for other conditions in other boroughs (local neighbourhoods) at no cost to the patients ‐*– '…but also those cooking classes are really really important because I know they do it for so many other conditions in other boroughs and it is free'* (SU‐C 9).

### 3.16 Subtheme 5 – Community and Policy Factors – Policy and Practice

Here a SCSU‐C explained the importance of engaging with the topic of nutrition in a more collaborative way within the community, but also on a strategic level that involves key stakeholders such as the British Dietetic Association (BDA), SCS, NHS England and the Clinical Commissioning Groups (CCGs) *– 'I think that um the SCS has got lots of connections with the CCG, with all the NHS England groups, so I think they are quite well placed to raise this issue to support people also'* (SU‐C 8). Equally, a SCSU‐C expressed the importance of sickle cell patients knowing how to advocate for themselves, for example writing to their local MP's and hospital boards to help raise awareness of nutrition and the quality of hospital food, and helping patients make better healthy choices – *'I think possibly people writing to their local MP and writing to the hospital board…to find better ways of giving people better healthy choices'* (SU‐C 2). In the context of policy and practice change, a SCSU‐C emphasised the need for nutrition to be part of clinical policy in the same way that people living with SCD access other non‐medicalised therapies and the need for access to be available to both inpatients and outpatients *– 'I think there is something about how to sort of push from a policy perspective and clinical policy, nutrition to be part of these non ‐medicalised therapies that we can have access to whether we are outpatients or inpatients'* (SU‐C 9).

## 4 Discussion

This study is the first attempt to understand the factors affecting nutrition integration in SCD within clinical dietetic practice in the UK and in doing so starts to address the paucity of qualitative studies in this area. Furthermore, this is the first study involving SCSU‐C participants to illuminate their views, knowledge and experiences of nutritional care providing helpful insights to define the current nutritional landscape in SCD. Clear evidence of nutritional knowledge and care gaps have been identified including an array of influencing factors compounding the lack of nutrition integration in sickle cell patient pathways, a potential health inequality affecting sickle cell patient's experience, access and outcomes of nutrition and service provision [12].

This study draws attention to the impact of the lack of integration of nutrition within standard care provision in SCD and within dietetic practice, adding to the marginalisation associated with SCD. The findings of this study thus bring the lack of nutrition service provision under scrutiny, providing evidence of the low level and quality of nutritional care provision available to the sickle cell patient population in the UK.

This oversight is in direct breach of the NHS Long term Plan [22] calling for the provision of personalised care [23], essential for people to have more control over their healthcare and provision and giving them a voice to advocate for their own health and nutritional needs. Moreover, the marginalisation of nutritional care provision in SCD is in breach of the NHS values and standards underscoring the provision of high‐quality care to all patients, including those living with SCD. This sentiment is echoed in the principles of the House of Care model [24] where care provision should be patient centred, with providers and patients working collaboratively and in partnership to provide care tailored to the needs of the patients, acknowledging the variances in sickle cell patients medical and nutritional care needs, as highlighted by a SCSU‐C. Although the benefits of the medical treatment modalities such as hydroxy carbamide are well established in SCD care, the findings of this study start to provide and build the evidence base that nutritional care is lacking in SCD, despite clear indications of the existence of the nutritional needs, risks and challenges of people living with SCD.

Nutrition was however identified as being ‘paramount’ and ‘fundamental’ to the health outcomes of participants and recognised as a ‘key part of keeping them well,’ especially for those who relied on nutrition as their main treatment option. The lack of nutrition care provision presented health risks to participants, who in the absence of nutritional care had to resort to self‐research inferring that they had to self‐diagnose and self‐manage their nutritional needs and concerns placing them at an increased risk of poor outcomes.

For example, a participant reported to being diagnosed with osteoporosis which came as a shock to them thus adding to their health risks due to the absence of nutritional care in SCD. This potentially means that other sickle cell patients may be at risk of the late diagnosis of nutritional problems, thus adding to the healthcare and cost burden to these patients and NHS respectively. Osteoporosis is not often recognised as a nutritional risk and people living with SCD may also be at high risk [25].

Many participants identified appetite suppression in line with existing scientific literature [19], as a key nutritional challenge and risk particularly when they are unwell or inpatients in hospital with already increased nutritional requirements. This adds to their existing nutritional risks and poor outcomes, length of stay and increased need for pharmaceutical interventions. Thus SCSU‐C participants were keen to have more opportunities, as part of their routine appointments, to ask questions and speak about their nutritional needs and concerns. While also wanting these conversations to happen earlier, and on an ongoing basis, thus providing pro‐active instead of reactive care thereby preventing and reducing their risk of developing nutritional problems in the future.

The participants highlighted the emphasis placed on drinking more fluid to prevent dehydration, a recognised trigger for vaso‐occlusive crisis, in line with existing health promotion advice [26], thus illuminating the limited view to the importance of nutrition as a management option in SCD care provision. Important to note is the increased life expectancy in SCD, as sickle cell patients are living longer due to improved medical care provision [27]. This means people with SCD may now experience different medical needs related to ageing, over and above the SCD‐related complications that in turn, may exacerbate their increased risk of morbidity, mortality, poor QOL and disability [28, 29, 30].

In addition to ageing related problems several other health related factors were identified by some of the SCSU‐C participants. These factors included being affected by high levels of inflammation, increased requirement for antioxidants and the impact of appetite suppression on their food intake especially when they were unwell, in concurrence with existing literature [10]. In addition, they also mentioned the importance of nutrition to support their mental health, a factor not previously reported in the literature. The timing of nutritional advice provided by consultants, was highlighted as a contributing factor to the under‐recognition of the importance of nutrition as a management option for patients with increased nutritional needs in SCD.

Several system related factors were also identified as evidence of the under‐recognition of the importance of nutrition, highlighting the absence of nutrition in the standard medical evaluations of patients, the fact that nutrition is seen to be ‘pushed aside’ and ‘not considered’ in their treatment. Moreover, there was a suggestion for nutrition to be considered in a more holistic way, similar to how physiotherapy, psychology and pain therapy was incorporated into the existing clinical standards of care for sickle cell patients [3]. Furthermore, gaps in nutrition knowledge and education were identified by a number of the SCSU‐Cs, a similar finding was identified in the seminal report by the All Party Parliamentary Group (APPG, 2021) [31] regarding the medical management of SCD. This indicates a growing need for nutrition education and training for doctors and nurses working in SCD to address the under‐recognition of nutrition in SCD care.

However, to gain more clarity on the nutritional care needs of sickle cell patients, a wider lens was needed to identify the array of influencing factors affecting nutrition and service provision, a gap in the existing sickle cell nutrition literature.

The study illuminated a range of influencing socio‐ecological factors, not previously explored in the existing scientific literature on the role of nutrition in SCD, potentially impacting on the nutritional needs, risks and challenges of sickle cell patients. These influencing factors highlighted in Table 2, reflect the micro, meso and macro level factors affecting sickle cell nutrition which in turn add to the complexity of the nutritional management of people living with SCD. Insert Table 2:

**Table 2:** Socio‐ecological factors affecting nutrition and service provision.

**Micro‐level**	**Personal internal factors:**
	Psychological, emotional, attitudes, beliefs, culture, stigma, religion, health symptomology
	**Personal external factors:**
	Environment, time, knowledge, family, culture, food traditions, peer support
**Meso‐Level**	Meeting unmet nutrition needs of patients
	Lack of nutrition service provision
	Lack of education, training and resources in nutrition in SCD
**Macro‐level**	**Structural social factors:**
	Ethnicity, race, social class, deprivation, poverty, education

Participants identified a range of internal and external biopsychosocial factors (see Table 2) influencing nutrition. The internal factors identified included how a person's ‘mood’ leads to ‘comfort eating’ and how some of the clinical symptoms like lethargy and tiredness, identified as the most common symptom in a recent international multi‐centre study [32], can affect a patient's ability to buy, prepare and eat well. Participants experiencing these clinical symptoms often relied on family members and their support to help them eat well during these challenging times. Participant's working environments may also play a role in their ability to make healthy food choices and eat at regular intervals, so this too needs to be considered as influencing factors. Equally, the timing of nutritional conversations to address knowledge gaps in sickle cell patients was identified as important and seen as way to raise awareness of the role of nutrition in SCD management.

The lack of nutritional care in SCD inspired a call for a more joint up approach to nutrition service provision. With the suggestion to developing joint clinics for both paediatric and adult sickle cell patients thereby supporting the nutrition education and training of doctors and nurses, to facilitate their skill development in nutrition. In addition, the lack of available nutritional information and resources were identified as an influencing factor, and moreover the need for nutrition education to be provided to all healthcare professionals.

Key community and policy level factors were also identified through the study which have not previously been considered in sickle cell nutrition. A key community factor related to the cost, affordability and availability of healthy food in the areas where sickle cell patients normally live, since many people with SCD are known to live in socio‐economically deprived areas [16, 33] and higher food costs can have a significant effect on the food choices made by patients, thereby impacting on their long‐term health and wellbeing. A potential disparity identified was regarding sickle cell patients having access to cooking classes, usually provided for other health conditions such as diabetes, in local London neighbourhoods, free of charge. This may help address the nutrition knowledge gaps identified amongst the sickle cell patient population in this study.

Moreover, the need for nutrition to be included in policy was also identified, calling on more collaborative engagement and discussion on the importance of the role of nutrition in the management of SCD. More collaboration was emphasised on a strategic level by a range of stakeholders including the BDA, SCS, NHS England and commissioning bodies, to address the lack of nutrition service provision in SCD, a key driver of the health inequality affecting patients experience, access and outcomes of nutrition. To facilitate this shift in policy, sickle cell patients need to be empowered to advocate for themselves by writing to their local MP's and hospital boards, and for nutrition to be part of healthcare policy to improve patients access to services both as inpatients and outpatients. For this reason, the holistic lived experience of people living with SCD is of paramount importance and the compartmentalisation of the various needs, for example the medical versus nutritional, should not be done in isolation but be considered holistically to support the integration of nutritional care into sickle cell patient pathways.

## 5 Strengths and Limitations

This research project, albeit a smaller sample size, with a cross‐sectional design is the first attempt to provide primary data based on the views, knowledge, direct and indirect lived experiences of SCSU‐Cs. These insights providing the first opportunity to better understand the medical and nutritional management landscape in SCD necessary to inform the development of future policy and practice guidance to support nutritional management and care in SCD.

The researcher was keen to identify and understand the influencing factors that may affect nutrition in SCD as this has not previously been explored in the existing literature. For this reason, a socio‐ecological framework was applied to the development of the focus group questions which could imply that focus group questions #4 to #10 in the topic guide may appear that nutrition is a problem in sickle cell in many different ways. The socio‐ecological framework enabled the researcher to explore factors such as nutrition and peer/family support and community‐based factors such as access to food in the patient's environment, important factors to consider in the context of identifying a person's risk of diet or disease related malnutrition, a known risk in SCD. Applying a socio‐ecological lens to the data collection has thus enabled the researcher to identify multiple influencing factors affecting nutrition in sickle cell and the need for a whole systems approach to service provision.

Furthermore, the more neutral framing of focus group questions #2 and #3 was primarily to establish participants experiences of nutritional care if any and the responses to these questions did identify nutrition to be a problem in sickle cell. This may have been influenced by the fact that the PIS introduced the topic of sickle cell nutrition, and this may also have influenced the participant responses. Overall, however, the development of the topic guide questions was informed by the existing scientific literature on the role of nutrition in sickle cell explicitly identifying the nutritional needs of sickle cell patients. This in turn informed the assumptions the researcher held about the existence of nutrition in sickle cell and a growing interest to explore patients' views, knowledge and experiences of nutritional care.

In addition, the qualitative data has highlighted areas and gaps in sickle cell nutrition that requires further development and exploration. This necessitates the need for further pilot studies and larger‐scale studies supported by a moderator to explore variability in the nutritional needs of patients and consider their disease severity and complications.

The lack of demographical data for the study participants left a gap in understanding the direct impact of ethnicity, age, employment status and socio‐economic standing of the study participants and how this affected their knowledge views and experiences of medical and nutritional management in SCD. However, some of these background insights have emerged through the participant responses, although collecting this information would have added to the rigour of the study since the research was conducted in a predominantly marginalised patient population. It is recommended that future studies include key demographic data, that extends beyond the gender of the participants, as it is hard to replicate study participants in qualitative studies.

Furthermore, the scaling and generalisation of the findings may be limited, and care is needed to acknowledge the variances in the disease severity of patients according to the genotype and phenotype manifestations and how these variances may affect the patients' nutritional needs and risks, whilst still facilitating their access to tailored nutritional care. Equally, there may be benefit from future studies being multi‐location, for example using the Heamoglobinopathy Coordinating Centres (HCC)s as a focal point to assess how well the findings of the study reflects others knowledge, views and perspectives on the medical and nutritional management of people living with SCD. Further studies could also begin to estimate the proportion of people who prioritise nutrition as a key part of managing their condition.

Recruitment bias was accounted for considering the study employed a purposive sampling technique with the support of gatekeepers who worked in a local sickle cell service where the researcher previously worked. The mention of nutrition in sickle cell in the PIS may also have influenced participants engagement in the study, however we cannot discount the participants knowledge, views or experiences of nutritional care or their reliance on nutrition as part of their management options in SCD. There is a possibility that the researcher's prior interest and assumptions about the existence of nutrition in sickle cell may also have influenced the participants contributions in the focus group. None the less, the possible skewing of the data is mitigated by using the four phased learning alliance methodology used in populations from black and ethnic minority backgrounds. This methodology helped to circumvent data bias and enhance the trustworthiness of the data allowing for multiple opportunities to verify the data collected giving both the researcher and the participants an opportunity to check the responses provided in the focus group.

The study therefore provided a timely opportunity to bring people living with sickle cell into the forefront of speaking about their knowledge, views and experiences of the medical and nutritional management of their condition. Additionally, the study gave the participants a voice to express the challenges and gaps in their care provision and changes they would like to see made to improve their access and outcomes of nutrition service provision.

## 6 Implications for Practice

The study findings are the first primary data to inform the development of tailored nutritional care provision and future policy and practice guidelines to support the nutritional management of people with SCD in the UK. There is well established literature supporting the biomedical evidence for nutrition in sickle cell, since the same clinical features of sickle cell (chronic haemolysis, vaso‐occlusion, impaired immunity) requiring medical management are the same clinical features that cause the nutritional problems in sickle cell. Some of the nutritional problems include chronic anaemia and fatigue, increased oxidative stress, chronic inflammation and increased risk of infection that increases a patient's risk of malnutrition [10].

In addition, the data is helping to understand the current nutritional management landscape in SCD that has contributed towards sickle cell nutrition now being recognised as an emerging specialism in the Profession of dietetics. Therefore, due diligence is needed to evaluate existing nutrition information, but equally also consider what can be learnt from other chronic conditions where patients have a risk of malnutrition to see what is relevant and applicable to the sickle cell patient population.

Currently there is a paucity of research on the nutritional management of SCD, however a positive step has been the inclusion of the inaugural chapter on ‘*Sickle cell and other haemoglobinopathies’* that was published in the 7th Edition of the Manual of Dietetic Practice [34]. The Manual of Dietetic Practice is recognised as the gold standard textbook to support dietetic practice and hence can inform the development of future specialised nutritional management plans, interventions and dietary programs tailored to the needs of the sickle cell patient population.

Furthermore, the study provides qualitative evidence of the reported nutritional needs, challenges and risks in the sickle cell patient population, heralding a call for more proactive care where sickle cell patients have more opportunity to ask questions about their nutritional needs, and be empowered towards better self‐management of their chronic condition.

This will require more effort to understand the nutritional needs of this patient population and enable the provision of tailored nutritional care and resources with more consideration given to what may be needed to integrate nutrition and dietetic care into standard patient pathways. The study findings are first steps to helping to define what is needed to improve patients access and outcomes to tailored nutritional care. Future improvements will benefit from taking a whole systems approach drawing on the influencing socio‐ecological factors identified in the study and key stakeholders to address the gaps in nutrition service provision and policy and practice guidance and change.

## 7 Conclusion

The ongoing invisibility of nutrition in SCD management needs to be challenged at both policy and practice levels, and the health outcomes of the patients living with SCD should become a core aspect of care provision in SCD.

Additionally, the existing nutrition knowledge and care gaps linked to the lack of nutrition service provision in SCD needs to be bridged to reduce the under‐recognition and lack of priority given to nutrition as part of the standard care of sickle cell patients, evidence of the marginalisation in SCD. Thus, nutritional care provision in SCD needs to be tailored and personalised to the specific nutritional needs of the patient, considering the multiple influencing factors affecting nutrition and service provision in SCD.

Therefore, the lack of evidence base in sickle cell nutrition, driving the current lack of policy and practice to support the nutritional care of patients, needs challenging. Fresh calls are needed to link research including the findings of this study to improve clinical practice in SCD [35] where the patient voice matters and allowed to influence the ‘changing sickle cell nutrition integration narrative’.

## Author Contributions

Claudine Matthews conceived the study. Claudine Matthews was responsible for the design, recruitment of participants and responsible for data collection, analysis and interpretation with support from David Smith and Michelle Hawkins. David Smith contributed to the choice of methodology. Claudine Matthews was responsible for the oversight of the study. All authors contributed to the review and writing of the article.

## Funding

The authors have nothing to report.

## Ethics Statement

The study was granted full ethical approval by Anglia Ruskin University (ESC‐SREP‐18‐334).

## Conflicts of Interest

The following authors, C.M., D.S. and M.H. declare no conflict of interest. A.B. declares researcher‐led grants from the National Institute for Health Research, Rosetrees Trust, MRC, INNOVATE UK, British Dietetic Association, British Association of Parenteral and Enteral Nutrition, BBRSC, the Office of Health Improvement and Disparities and NovoNordisk. AB reports honoraria from Novo Nordisk, Lilly, Office of Health Improvement and Disparity, Johnson and Johnson and Obesity UK outside the submitted work and is on the Medical Advisory Board and shareholder of Reset Health Clinics Ltd.

## Transparency Statement

The lead author affirms that this manuscript is an honest, accurate and transparent account of the study being reported. The lead author affirms that no important aspects of the study have been omitted and that any discrepancies from the study as planned have been explained.

## Supporting information

Supporting File

## Data Availability

The data that support the findings of this study are openly available in ARU.Figshare.com at https://hdl.handle.net/10779/aru.24925884. The data that support the findings of this study are openly available in the figshare repository at https://aru.figshare.com/articles/thesis/Co-developing_a_health_literacy_framework_to_integrate_nutrition_into_standard_care_in_sickle_cell_disease/24925884?file=43871958.
