# Supplementary material for: Changing the Sickle Cell Nutrition Integration Narrative: Qualitative Perspectives From Sickle Cell Service Users/Carers About Nutritional Care
Source: J Hum Nutr Diet. 2026 Jul 14;39(4):e70303. doi: 10.1111/jhn.70303 (PMC13370134; doi:10.1111/jhn.70303)
Supplement: Supplementary file 1 — Supporting File [file JHN-39-0-s001.docx]

**Supplementary Material: Phase one Topic Guide questionnaire**

**Research sickle cell service user/carer question schedule:**

1. What do you know about the main clinical problems of sickle cell and how it is managed?
2. What role do you think does nutrition play in the management of your sickle cell?
3. What is your understanding of optimum nutrition and describe what you think are the main nutrition needs of sickle cell patients across the **life span (infants, children, adolescents, adults, pregnancy)?**
4. What are some of the main nutrition challenges you or those you care for face and how do you manage it?
5. How do you think service providers can help to identify, assess and manage your nutrition needs more effectively?
6. In your opinion what are some of the personal (**your** **social, emotional, psychological, physical, nutrition knowledge**) factors that may affect your food choices/beliefs/habits/behaviours? – why do you think that? – what are some of the implications?
7. What are some of the interpersonal (**interactions with peers, family, service providers**) factors that may influence your food choices/beliefs/habits/behaviours? – Why do you think that? – what are some of the implications?
8. What may be some of the organisational (t**he places where you receive health, social care, education**) factors that may be affecting your nutrition needs – (**thinking about how nutrition is assessed, monitored and managed**)? Why do you think that? – what are some the implications?
9. What may be some of the community level factors (**areas where you live, work, have leisure activities, shop**) that may be affecting your nutrition needs – (**thinking about your access to healthy food options, nutrition educational resources, healthy eating advice, tailored nutrition activities, cooking lessons etc**)? Why do you think that? – what are some of the implications?
10. Taking one factor at a time – (**personal, interpersonal, organisational, and community level factors**) – what suggestions do you have to improve the impact these factors may have on your nutritional needs?
